# Longterm quality of life after oncologic surgery and microvascular free flap reconstruction in patients with oral squamous cell carcinoma

**DOI:** 10.4317/medoral.21111

**Published:** 2016-03-31

**Authors:** Andre Peisker, Gregor-Franziskus Raschke, Arndt Guentsch, Korosh Roshanghias, Francy Eichmann, Stefan Schultze-Mosgau

**Affiliations:** 1 MD, DMD. Department of Cranio-Maxillofacial & Plastic Surgery, Jena University Hospital, Erlanger Allee 101, 07747 Jena, Germany; 2 MD, DMD, PhD. Department of Cranio-Maxillofacial & Plastic Surgery, Jena University Hospital, Erlanger Allee 101, 07747 Jena, Germany; 3 DMD, PhD, MHBA. Department of Surgical Sciences, Marquette University, School of Dentistry, Milwaukee, Wisconsin 53201-1881, USA; 4 DMD. Department of Cranio-Maxillofacial & Plastic Surgery, Jena University Hospital, Erlanger Allee 101, 07747 Jena, Germany; 5 DMD. Department of Cranio-Maxillofacial & Plastic Surgery, Jena University Hospital, Erlanger Allee 101, 07747 Jena, Germany; 6 MD, DMD, PhD. Department of Cranio-Maxillofacial & Plastic Surgery, Jena University Hospital, Erlanger Allee 101, 07747 Jena, Germany

## Abstract

**Background:**

Quality of life (QoL) has become increasingly important in cancer treatment. It refers to the patient’s perception of the effects of the disease and therapy, and their impact on daily functioning and general feeling of well being.

**Material and Methods:**

n this prospective study, a total of 100 patients treated at our institution, completed the European Organization for Research and Treatment of Cancer (EORTC) QLQ-C30 questionnaire and the specific EORTC QLQ-H&N35 module. The questionnaires were distributed to the patients between 12 and 60 months postoperatively.

**Results:**

Global QoL score was 58.3 and mean score for functioning scale was 76.7. Fatigue (28.7 ± 26.1), followed by financial problems (27.7 ± 33.5), insomnia (26.7 ± 34.5) and pain (26.3 ± 29.9) had highest symptom score on QLQ-C30. Fatigue (r=-0.488), insomnia (r=-0.416) and pain (r =-0.448) showed highest value for significantly negative correlation to global QoL. In the H&N35 module, restriction of mouth opening (43.3 ± 38.6), dry mouth (40.7 ± 36.9), sticky saliva (37.3 ± 37.1) and eating in public (33.8 ± 31.9) were the four worst symptoms. Swallowing problem (r=-0.438), eating in public (r=-0.420) and persistent severe speech (r=-0.398) ranked as the three worst symptoms with highest value for significantly negative correlation to global QoL.

**Conclusions:**

Longterm QoL after oncologic surgery and microvascular free flap reconstruction in patients with oral cancer is satisfactory. Measuring QoL should be considered as part of the evaluation of cancer treatment.

**Key words:**Longterm quality of life, oral cancer, oncologic surgery, microvascular free flap reconstruction.

## Introduction

Generally, the evaluation of cancer treatment is focused on the survival rate, local control rate, or complication rate. The main disadvantage is that these endpoints were usually assessed from the physician´s points of view. Therefore quality of life (QoL) has become increasingly important in cancer therapy. It refers to the patient’s perception of the effects of the disease and the impact of the operation on the patient’s daily functioning. Due to debilitating problems with swallowing and speech as well as the psychological effects of loss of function and change in body image, QoL present a decisive role in patients with oral cancer (1). Numerous of well-validated QoL instruments are established in the field of oncology. Especially, the European Organization of Research and Treatment of Cancer Quality of Life Core Questionnaire C30 (EORTC QLQ-C30) and the Quality of Life Head and Neck module (EORTC QLQ-H&N35) were frequently used (2,3). Prospective QoL studies on patients suffering from head and neck cancer with more than 12 months of follow-up are rare in the current literature (4,5). The aim of this prospective study was to evaluate long-term QoL after oncologic surgery and immediate microvascular reconstruction in patients with oral cancer.

## Material and Methods

- Study population

The prospective study was initiated after the local ethics committee of the Jena University Hospital gave its approval. Written informed consent was obtained from all participants. All patients had histological confirmed diagnoses of oral squamous cell carcinoma (OSCC) and underwent surgery at the Jena University Hospital with curative intent. After intraoral radical resection and lymph node surgery in accordance with preoperative clinical and radiological examination, all patients received immediate microvascular free flap reconstruction.

- Assessment of QoL

Patients completed the German versions of the EORTC QLQ-C30 and the EORTC QLQ-H&N35 at between 12 and 60 months after treatment.

The EORTC QLQ-C30 consisted of five functional scales (physical function, cognitive function, role function, emotional function and social function), three symptom scales (fatigue, emesis and pain), one scale for the overall health status/QoL and six single items (breathing, sleep disorders, appetite loss, constipation, diarrhea and economic sequelae) (3).

The EORTC QLQ-H&N35 comprises seven multi-item scales (pain, swallowing, senses, speech, social eating, social contact, sexuality) and eleven single items. All scales and single item variables were transformed into a score from 0 to 100 (2).

A high score for the functioning scale and for the global QoL scale represents a better level of functioning, whereas higher levels in the symptom scales or the single-item scales of the EORTC QLQ-C30 and the H&N module denotes a high level of symptoms or problems.

- Statistical analysis

Statistical analyses were conducted using the SPSS/PC statistical program (version 22.0 for Windows; SPSS, Inc., Chicago, IL). Using the Pearson’s correlation coefficient (r), we investigated the potential relationships between QoL global score and general, head and neck symptoms.

In general, r > 0 indicates positive relationship, r < 0 indicates negative relationship, while r = 0 indicates no relationship (or that the variables are independent and not related). The strength of relationship is strong for value of r -1.0 to -0.5 or 0.5 to 1.0, moderate for value of -0.5 to -0.3 or 0.3 to 0.5, weak for value of -0.3 to -0.1 or 0.1 to 0.3 and none or very weak for value of -0.1 to 0.1. It is well recognised that a correlation between two variables exists when r is superior to 0.3 and that this correlation increases as r approaches 1 (6).

Levels of statistical significance have been calculated at the 5% level of probability (*p* <0.05).

## Results

A total of 100 patients (69 male, 31 female), mean age 60.1±11.2 years (range 40-83 years), were enrolled in this study. The location of the tumour and the stage of disease are shown in  Table 1. In the majority of cases (42 %), the tumour was located on the floor of the mouth and 67 out of 100 patients presented with an early stage I/II of disease. The radial forearm flap was most commonly used ( Table 1). In all, adjuvant therapy was indicated in 74 cases. 59 Patients underwent postoperative radiotherapy and further 15 patients received postoperative radiochemotherapy.

**Table 1:**
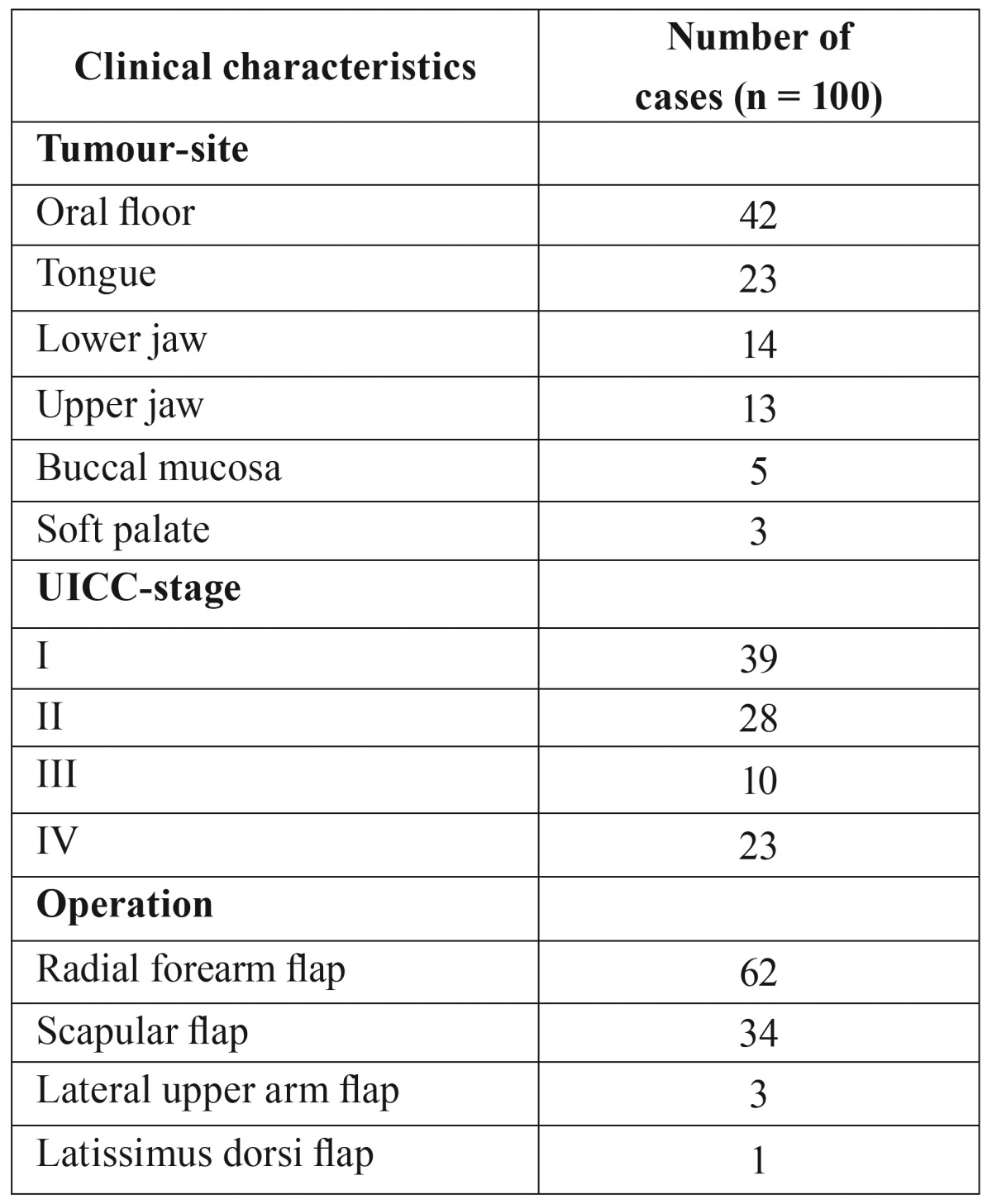
Clinical characteristics of the 100 patients.

- EORTC QLQ-C30

The results of the functional scales are shown in  Table 2. The mean QoL global score was 58.3 and the mean score for functioning scale of 76.7.

**Table 2:**
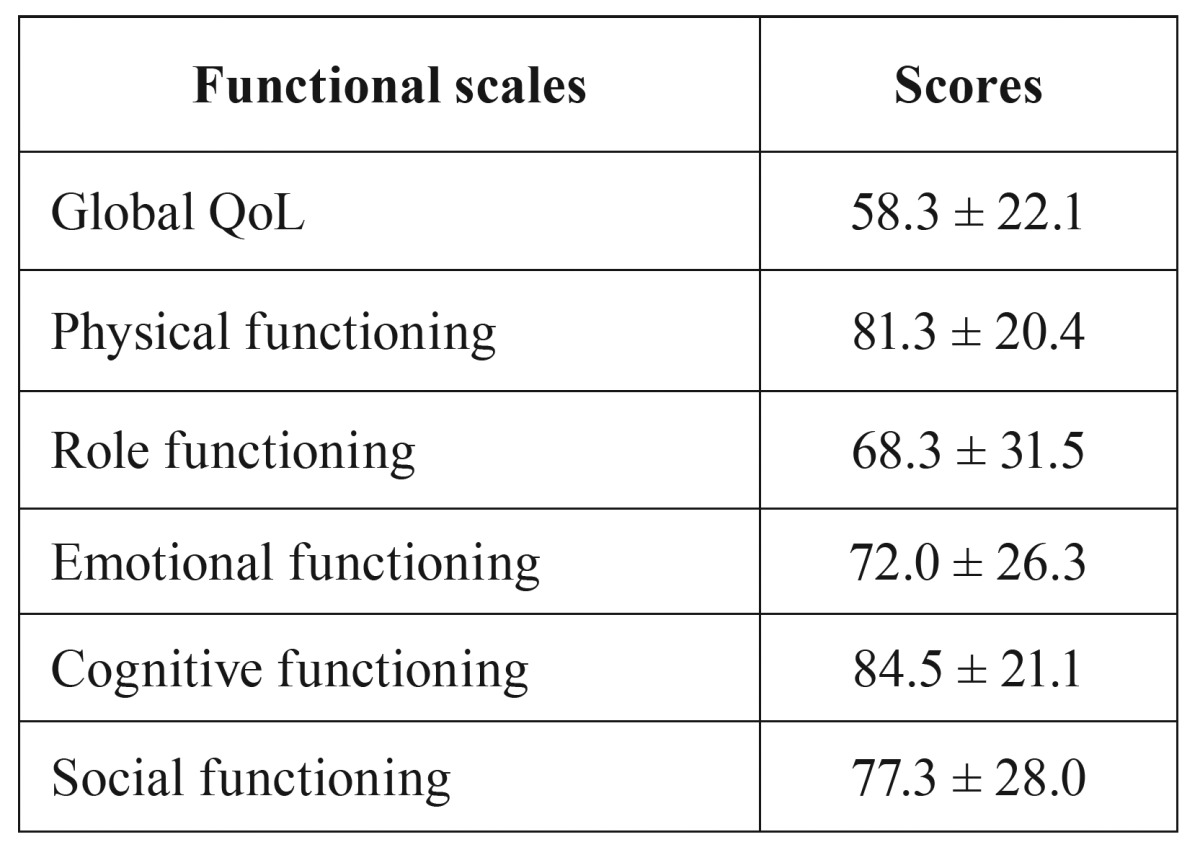
Results of the QLQ-C30 overall health status and functional scales.

The results of the general symptom scales and the bivariate analyses between global QoL score and general symptoms are shown in  Table 3. Fatigue (28.7 ± 26.1), followed by financial problems (27.7 ± 33.5), insomnia (26.7 ± 34.5) and pain (26.3 ± 29.9) were the main general complaints.

**Table 3:**
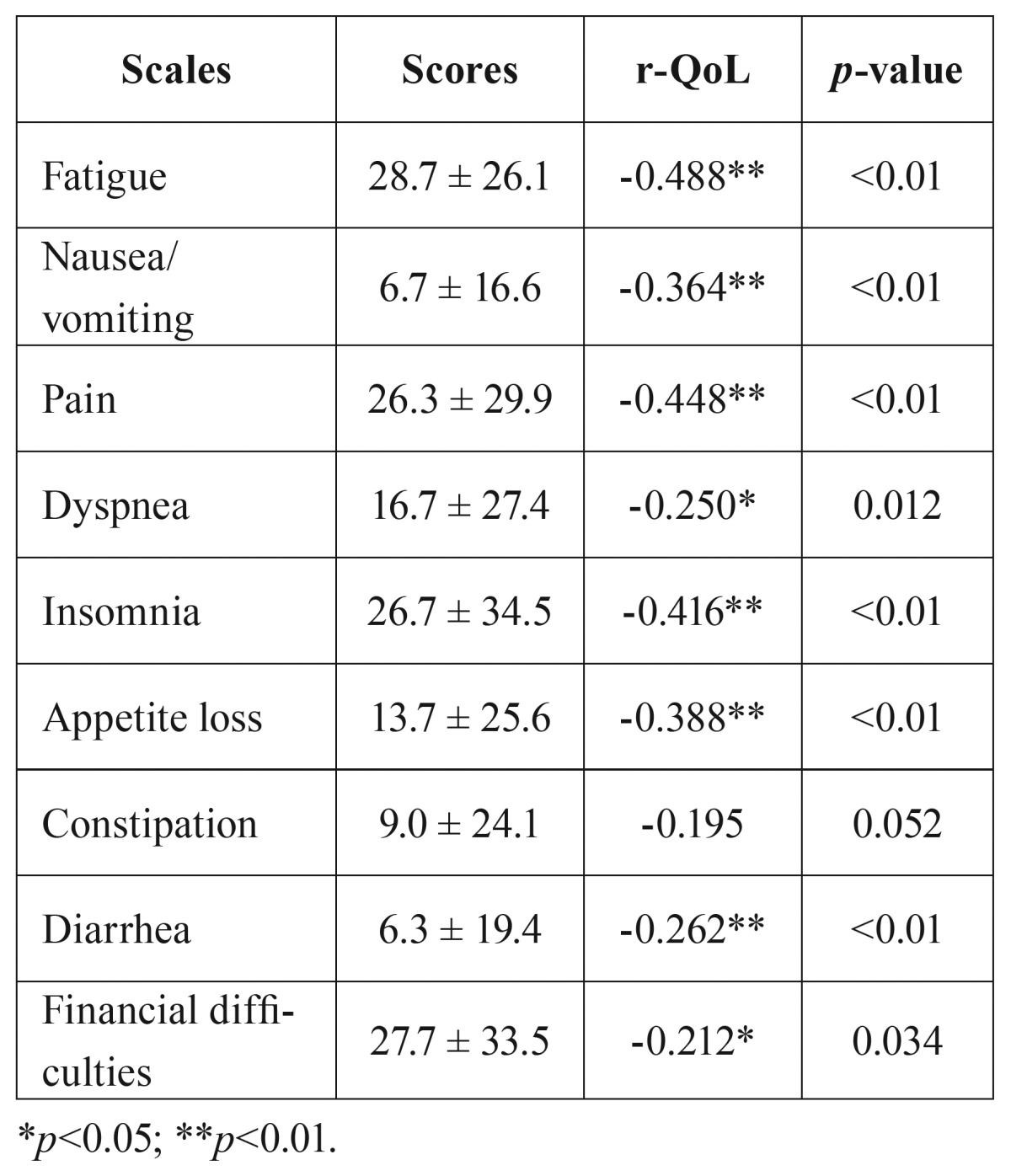
Results of the QLQ-C30 and the bivariate analyses between QoL score and general symptoms (r-QoL).

- Global QoL score was 

• significantly (*p*<0.05) negative correlated with dyspnea (r=-0.250) and financial difficulties (r=-0.212) 

• very significantly (*p*<0.01) negative correlated with fatigue (r=-0.488), nausea (r=-0.364), pain (r =-0.488), insomnia (r=-0.416), appetite loss (r=-0.388), and diarrhea (r=-0.262).

However, global QoL score was not correlated with constipation.

- EORTC QLQ-H&N35

The results from the EORTC QLQ-H&N35 scales and items are shown in  Table 4. Restriction of mouth opening (43.3 ± 38.6), dry mouth (40.7 ± 36.9), sticky saliva (37.3 ± 37.1) and eating in public (33.8 ± 31.9 ) ranked as the four worst symptoms.

**Table 4:**
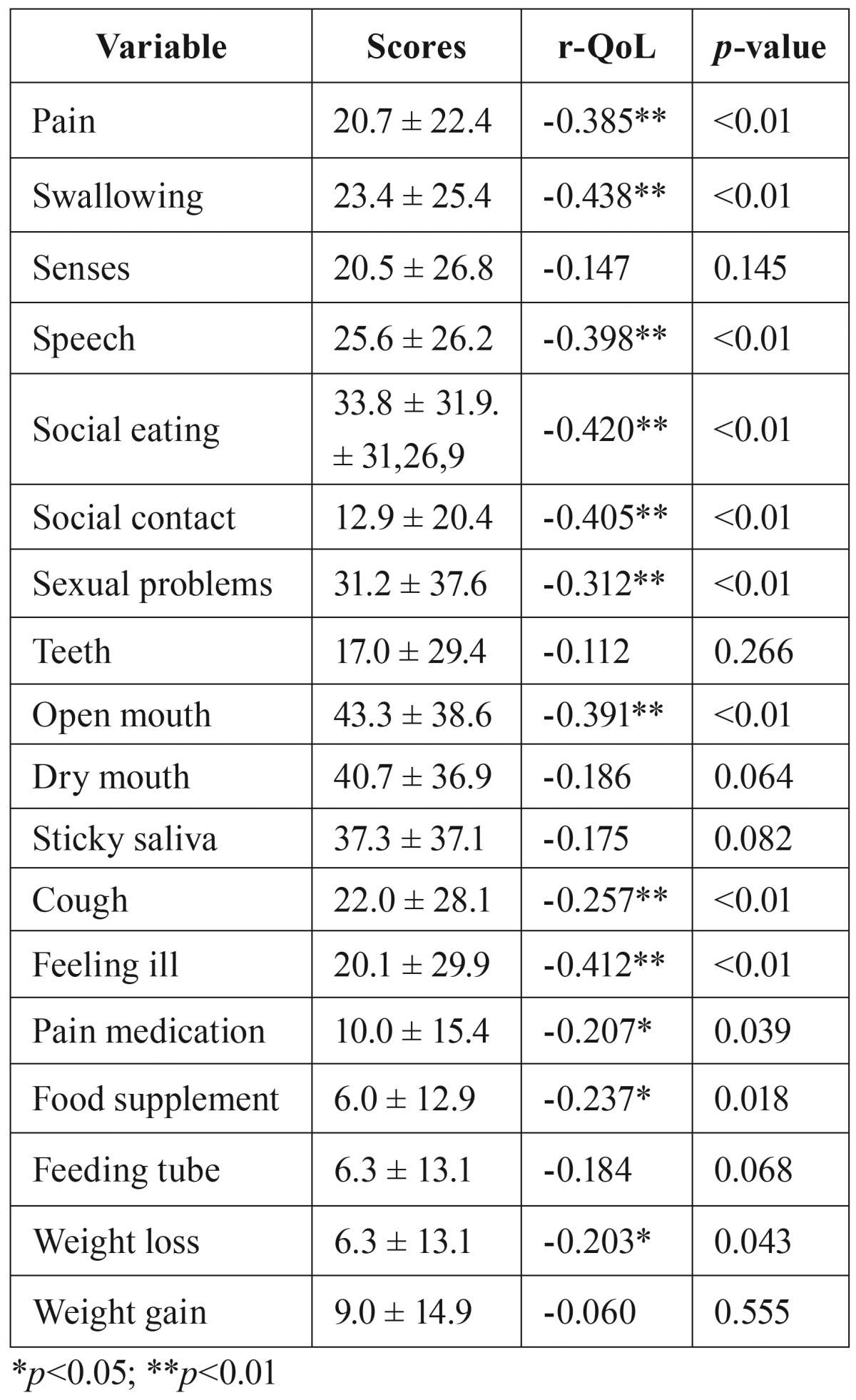
Results of the QLQ-H&N35 and the bivariate analyses between QoL score and symptoms (r-QoL).

- Global QoL score was 

• significantly (*p*<0.05) negative correlated with problems with pain medication (r=-0.207), food supplements (r=-0.237) and weight loss (r=-0.203).

• very significantly (*p*<0.01) negative correlated with pain (r=-0.385), swallowing (r=-0.438), speech (r=-0.398), social eating (r=-0.420), social contact (r=-0.405), sexual problems (r=-0.312), open mouth (r=-0.391), cough (r=-0.257) and feeling ill (r=-0.412).

There was no correlation between global QoL and problems with senses, teeth, dry mouth, sticky saliva, feeding tube and weight gain.

## Discussion

QoL has become a increasingly important in the assessment of any therapy in oncology. There is a rapidly increasing number of published studies investigating QoL in patients with head and neck cancer (2,5-8). Meanwhile numerous of well-validated QoL instruments are now available which have been categorized in three types of methods.The first category includes the generic type, e.g. the Short Form-36 (SF-36), the second the cancer specific type, e.g. the Functional Assessment of Cancer Treatment (FACT-G), the EORTC QLQ-C30, and the third the cancer site-specific type, e.g. the head and neck modules in EORTC QLQ-H&N35, and FACT (FACT-HN) (2,3,9-11).

Microvascular free tissue transfers have been used routinely for head and neck reconstruction in order to improve the functional and esthetic outcomes (6). Therefore, the aim of the present study was to evaluate long-term QoL after surgery and immediate free flap reconstruction in patients with OSCC.

We used a general QoL questionnaire, adapted to all cancer patients, the EORTC QLQ-C30, and a specific questionnaire, the EORTC QLQ-H&N35 which were completed at between 12 and 60 months after treatment.

Studies showed that surgical treatment of oral cancer led to a temporary deterioration of QoL issues. However, the levels of these scores improved until the end of the first postoperative year (7,12). In addition, there are not any further changes in QoL in the follow years after treatment (13). Therefore, it is generally accepted that QoL at one year post-treatment is a good indicator of QoL at long term in disease-free head and neck cancer patients (14,15).

With a global QoL score close to 60 %, long-term QoL of our patients was comparable to the study from Wan Leung *et al.*(1). Meanwhile other authors refer to a global QoL score close to 70 % (6,16). Furthermore the mean score for functioning scale of 76.7 % was similar to the results from Pierre *et al.* with a mean score of 82.5 % (6). Overall, persistent fatigue (28.7 ± 26.1) , followed by financial problems (27.7 ± 33.5), insomnia (26.7 ± 34.5) and pain (26.3 ± 29.9) were the main general complaints of our patients and significantly negative correlated with global QoL (fatigue: r=-0.488, financial problems: r=-0.212, insomnia: r=-0.416, pain: r=-0.448). Pierre *et al.* and Wan Leung *et al.* corroborated these results, as they also refer that fatigue and insomnia are the main general symptoms (6,1).

In the H&N35 module restriction of mouth opening (43.3 ± 38.6), dry mouth (40.7 ± 36.9), sticky saliva (37.3 ± 37.1) and eating in public (33.8 ± 31.9) denote a high level of problems. The limitation of mouth opening can be induced by surgical treatment (17,18). Furthermore the adjuvant radiotherapy is responsible for the limitation of mouth opening long term after treatment and for salivary dysfunction (19-22). Swallowing problem (r=-0.438), persistent severe speech (r=-0.398) and eating in public (r=-0.420) were significantly negative correlated with global QoL. These complaints should be properly identified in order to rehabilitate and nutritionally support patients. Therefore a nutritionist and a rehabilitation therapist should be part of the multidisciplinary team planning the care of these patients (8). Furthermore, studies reported that that QoL of patients with OSCC can benefit from psychological group therapy and psychoeducational treatment (23,24).

Feeling ill (r=-0.412) and pain (r=-0.385) were also significantly negative correlated with global QoL. Chronic pain is often a consequence of neck dissection and common after surgical treatment (25). Terrell *et al.* reported that neck dissection is associated with significant decline in global QoL (26). Postoperative rehabilitation should incorporated in the standard management in these patients. Pfister *et al.* found a significant reduction in pain and dysfunction in patients undergoing neck dissection who received weekly acupuncture versus usual care (27).

The design of the current study has some limitations. We did not subclassify the patients according to the adjuvant therapy. However, the size of the subgroup of patients with surgery alone and postoperative radiochemotherapy was too small and inhomogeneous for comparison of surgical and combined therapy. Furthermore, the focus of this study was to evaluate the effect of immediate microvascular free flap reconstruction on long-term QoL.

## Conclusions

The results of the present evaluation with the EORTC instruments show that longterm QoL after tumor resection and immediate microvascular free flap reconstruction in patients with OSCC seems to be acceptable. The highest symptom score on QLQ-C30 was for fatigue, followed by financial problems, insomnia and pain. In the H&N35 module, restriction of mouth opening, dry mouth, sticky saliva and eating in public ranked as the four worst symptoms. Measuring QoL should be considered as part of the evaluation of cancer treatment.
